# The Quality of Life of Mustard Gas Victims: A Systematic Review

**Published:** 2017

**Authors:** Mojtaba Satkin, Mostafa Ghanei, Abbas Ebadi, Sahar Allahverdi, Mahdi Elikaei

**Affiliations:** 1 Behavioral Sciences Research Center, Baqiyatallah University of Medical Sciences, Tehran, Iran,; 2 Chemical Injuries Research Center, Baqiyatallah University of Medical sciences, Tehran, Iran,; 3 Behavioral Sciences Research Center, Nursing Faculty of Baqiyatallah University of Medical Sciences, Tehran, Iran.

**Keywords:** Chemical agent, Chemical injury, Mustard gas, Quality of life, Systematic review

## Abstract

**Background:**

Today, a host of veterans who were exposed to mustard gas suffer from substantially poor quality of life (QoL). However, factors that influence these patients’ QoL have not been yet scrutinized. QoL is deemed as a crucial construct that demands careful attention during evaluation as well as intervention. The present study aimed to delve into the physical, mental, and social factors that affect the QoL of mustard gas victims.

**Materials and Methods:**

All the physical, mental, and social parameters that influence the QoL of mustard gas victims were scrutinized through a systematic review. We searched for Persian and English scientific databases, i.e., PubMed, Scopus, and Google Scholar, and national databanks, namely SID, IranMedex, and Magiran to identify studies related to chemical victims conducted up to the end of 2015. Next, the quality of 21 articles and studies were assessed using the checklist of the National Institute of Health (NIH), and subsequently, 13 articles were selected for the stages of data extraction and analysis.

**Results:**

Findings revealed that, among the physical factors, coexistence of several medical conditions caused by chemical injury and the severity of the chemical injury had the greatest impact on the QoL of chemically injured veterans. Besides, suffering from psychological and neurological disorders, along with educational level and employment status, were the most influential psychosocial parameters that influenced veterans’ QoL.

**Conclusion:**

The review conducted herein identified the physical and psychosocial factors affecting the QoL of mustard gas victims. In fact, it is the first to present a large collection of descriptive information on QoL contributors in a systematic and orderly fashion.

## INTRODUCTION

Exposure to different hazardous chemical agents, specifically gases, may result in various adverse acute and chronic medical consequences (1). Such agents can also be utilized in wars. For instance, chlorine gas that was first employed by the German army during World War I (2). The most widely used gas during WWI was Sulfur Mustard (SM), a vesicant agent initially introduced by the German army (3). After WWII, Iran has been the main victim of chemical attacks. Nearly 378 chemical attacks have been carried out during the Iran–Iraq war. There are more than 40,000 chemical victims in Iran, who were exposed to different blistering, blood, or nerve agents such as sulfur mustard (4).

Iranian researchers have found numerous late complications among victims of chemical attacks, including a wide range of respiratory, ocular, dermal, psychological, hematological, immunological, gastrointestinal and endocrine problems, all of which can influence the QoL of the exposed victims (5). Physical diseases and injuries caused by such agents affect various organs and tissues, including the skin, eye, or respiratory and gastrointestinal systems. Common exposure symptoms include severe forms of irritations, over activation of different secretory glands, coughs, etc. Respiratory problems such as acute or chronic Bronchiolitis and Bronchiectasis are some of the most important chronic disabilities (6, 7). These continuous irritations and exacerbations decrease the QoL of patients significantly. In addition to the acute phase symptoms of contaminations, chronic phase symptoms and signs highly impact the QoL of victims (8). Brain damage or severe organ injuries can influence the mental health of victims, which may be accompanied by familial, social, and even economic problems (9, 10).

According to different studies, chemical victims’ QoL scores were lower as compared to those of normal civilians (11). Research and therapeutic approaches to chemical victims’ complications are different from the approaches to other conventional lung diseases (12). Despite the positive effects of standard therapeutic protocols that are used for usual lung diseases such as asthma and COPD, such procedures are not suitable for chemically injured victims because of the fast emerging side effects. Thus, as lung exposure to mustard gas does not fit the signs and symptoms of any specific disease, it was named as “Mustard Lung” (13). This phrase uniquely represents the specific nature of such lung symptoms (14). Similarly, the QoL of such patients involves different and exceptional aspects according to altered signs and therapies. Therefore, we coined the term “Mustard Quality of Life (MQoL).” In this regard, improving the QoL of chemically injured veterans ought to be synched with the specific and unique nature of the morbidity, based not only on the physical, but also on the spiritual, psychological, cultural, social, familial, and economic aspects of QoL (15).

This study tries to combine all available data on the different aspects of the QoL of chemically injured veterans in order to facilitate further detailed and targeted studies on similar cases. Native articles have been frequently referenced in this study because, as previously mentioned, Iran is still the main victim of vast utilization of chemical agents, and nearly all acute-chronic studies have been established and initiated by Iranian scientists. The purpose of this study was to determine the diverse aspects of the physical and psychosocial factors affecting the QoL of victims exposed to mustard gas. Thus, the present explored to what extent the QoL of chemical victims is influenced by physical and psychosocial factors.

## MATERIALS AND METHODS

### Study design

This systematic review was conducted according to the guidelines of the Center for Reviews and Dissemination, York University (16). All studies pertaining to QoL of people who were injured by sulfur mustard were examined carefully. As specified in the guidelines, the review comprised the following procedural elements: 1) the review question, 2) strategy to search databases, 3) inclusion criteria for studies, 4) tools for quality assessment and study appraisal, 5) study selection based on eligibility, 6) data extraction and analysis, and 7) data synthesis.

To identify other systematic reviews with objectives similar to those of the current study, the Cochrane Database of Systematic Reviews was searched for full-texts of new systematic reviews, and no similar systemic reviews were identified (17). Next, relevant literature was searched for using keywords and a search formula. For devising a thorough search formula for the present systematic review, two trained scientists, one in the branch of psychology and another in medicine, counseled the research team to retrieve relevant articles better. Identified keywords were first used individually, following which, they were used along with the search formula. The review team searched both Persian and English databases while setting no time limit for relevant pieces of information. They retrieved all articles from the past up to the end of 2015. Meanwhile, useful information was stored on Endnote X7.

As mentioned above, Iran has sustained the most extensive damage caused by chemical attacks since WWII (4). Therefore, there is a great magnitude of relevant studies in Persian, which, along with English articles underwent rigorous inspection of their titles and abstracts. The keywords “chemical veterans,” “chemical warfare,” “chemical warfare agents,” “chemical weapon,” “chemical injuries,” “quality of life,” “mustard,” and “mustard gas” were searched in Scientific Information Database (SID), Database of Iranian Medical Sciences Articles (IranMedex), and Database of The Iranian Press (Magiran). Moreover, the review team manually searched specialized journals within the scope of chemical victims, i.e., the Iranian Journal of War and Public Health, Journal of Military Medicine, and Journal of Behavioral Sciences. Subsequently, three scientific databases, i.e., PubMed, Scopus, and Google Scholar were searched. In PubMed, an advanced search was conducted via a formula of identified keywords. In the Scopus database, an advanced search of titles and abstracts was conducted using the formula of identified keywords. Finally, Google Scholar was searched for relevant studies using the previously used formula of identified keywords.

### Selection criteria

Eligible studies comprised all articles which revolved around the assessment of QoL of chemical victims who met the inclusion criteria of the current systematic review. The review team narrowed the search area down to quantitative studies which covered human issues and dealt with problems of the mustard gas victims themselves (not their spouse, children, or family). Additionally, only original articles were considered for inclusion. Therefore, other sources of information, such as books, review articles, journals, and reports that did not fulfill the inclusion criteria like conciseness and capability to undergo quality assessment were excluded from the assessment. Next, the titles and abstracts of the retrieved articles were separately assessed by two referees (MS & MG). Here, studies that did not clearly satisfy the inclusion criteria were excluded from the systematic review. On completion of this preliminary assessment, the full text of the remaining articles was retrieved so that the referees would perform eligibility assessment once more. Those articles that were subjects of controversy between the referees were referred to a senior scholar (AE) to judge their eligibility.

### Quality assessment

In this study, the studies that could answer the present research question were examined. These articles underwent a critical assessment via a checklist provided by the National Institute of Health (NIH). The checklist comprises 14 items for the quality assessment of articles (18), which was later modified in accordance with the research objectives and question, as well as the instructions of York University. After the quality assessment, articles obtaining 60% of the total score were subjected to subsequent analysis.

In this step, the required data on the dimensions of QoL and its influential factors among mustard gas victims were extracted. The data included demographic characteristics, chemical injury-related characteristics, instruments, educational level, and physical as well as psychosocial dimensions affecting the QoL of mustard gas victims.

## RESULTS

The current systematic review constituted the four main stages of identification, screening, quality assessment, and analysis (16). In the first stage, searching Persian and local databases, the review team found 1145 records in SID, 2258 records in IranMedex, and 829 records in Magiran. Besides, on searching specialized Persian journals related to chemically injured veterans, the authors identified 40 records in the Iranian Journal of War and Public Health, 123 records in the Journal of Military Medicine, and 5 records in the Journal of Behavioral Sciences. The advanced search of international databases via identified keywords yielded 131 records in PubMed, 205 records in Scopus, and 61 records in Google Scholar. In sum, 4797 records, including articles, books, and reports, were identified at this stage, which were simultaneously stored in EndNote X7. From these, 960 duplicates were excluded; thus, 3837 records remained. In the second stage, the referees assessed the titles and abstracts of the retrieved articles with reference to the present research question, and 77 articles were retained for further analyses.

In the third stage, full-text versions of the remaining articles were retrieved to assess their eligibility. Thus, 56 articles that did not meet the necessary requirements were excluded from the systematic review. Accordingly, 21 eligible studies that conformed to the present research question and met the inclusion criteria were subjected to through quality assessment using the checklist developed by NIH. The quality assessment revealed that 4 articles did not have the essential data related to the current research question or did not employ the cross-sectional design. Four other articles failed to achieve the minimum required score on the checklist. Therefore, only 13 articles were used for the subsequent stages of data extraction and analysis. Besides, to gather additional facts and details needed for the current study, the review team communicated with the corresponding authors of two articles.

### Included studies

Data regarding demographic and chemical injury-related characteristics (Table 1), and physical and psychosocial factors affecting the QoL of the sample (Table 2) were extracted. The average age of the mustard gas victims was 44.22 years, and they were aged between 41.18 (5.6) and 48.08 (7.8) years. The total sample population comprised 2555 people. In addition, information on “educational level” was provided in 10 articles, on “employment status” in 9 articles, on “rate of chemical injury” in 4 articles, on “mean time since last exposure” in 9 articles, and on “frequency of exposure” in 3 articles.

**Figure:**
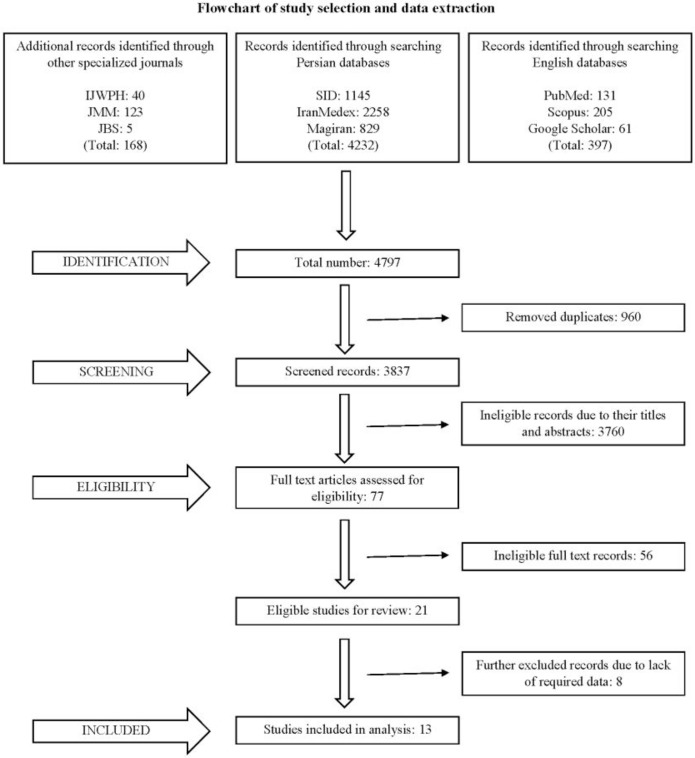


**Table 1.** Demographic characteristics and chemical injury-related characteristics

**Author**	**Year**	**n**	**Age (SD) yrs**	**Educational level**	**Employed**	**Mean rate of Chemical injury**	**Mean time since last exposure (SD) yrs**	**Frequency of exposure**
Abbasi (15)	2012	260	45.36(15.27)	83.8%≥12 16.2% >12	38.1%	NR	NR	NR
Ebadi et al (19)	2014	242	44.12(4.9)	40.1% >12	NR	28.71%	23.05(1.48)	3< 71.1%
Mousavi et al (20)	2009	147	44.8(8.6)	71.4% > 12 28.6%>12	25.2%	NR	21.6(1.2)	1= 66.3%, 1< 33.7%
Jafari et al (21)	2012	292	48.08(7.8)	49%> 12 47%≥12	26.4%	NR	NR	1< 27.6%
Mousavi et al (22)	2011	414	46.1(8.5)	50%≥12 50% >12	27.8%	NR	20	NR
Attaran et al (23)	2006	43	42.5(7.4)	NR	NR	NR	17.2 (4.4)	NR
Arefnasab et al (24)	2013	41	48	NR	100%	NR	26	NR
Mehdizadeh et al (25)	2011	93	44.03(6.56)	46.2%> 12 53.8%≥12	97.8%	36%	14	NR
Tavallaie et al (26)	2006	383	41.18(5.6)	23.4%> 12 66.6%≥12	90%	24.58%	20	NR
Tavalie et al (27)	2008	163	40(14.61)	92%≥12 8% >12	NR	17%	15	NR
Panahi et al (28)	2008	125	44.3(8.0)	NR	NR	NR	19	NR
Biat Saeed et al (29)	2104	120	47.14(4.5)	61%≥12 39% >12	35%	NR	NR	NR
Berahmani et al (30)	2004	232	41.05 (11.1)	91.4%≥12 8.6% >12	62.9	NR	NR	NR

**Table 2.** Physical and psychosocial factors effective in QoL of mustard gas victims

Author (reference)	Year	Measures		Influential factors

			Physical	Psychosocial
Abbasi (15)	2012	Abbasi’s specific Tool	-Co-existence of pulmonary, ocular, dermal and mental disorders (⇩)	-Anxiety (⇩) -Destruction of self-confidence (⇩)
Ebadi et al (19)	2014	SF-36	-Co-existence of pulmonary, ocular, dermal and mental disorders (⇩) -Number of injured organs (⇩) -Severity of chemical injury (⇩) -Poor physical health (⇩)	-Religious beliefs (⇧) -Low level of general health (⇩)-Poor emotional role (⇩) -High level of education (⇧)
Mousavi et al (20)	2009	SF-36	-Lengthy continuation of chemical injury (⇩) - Lack of sports activities(⇩) -Poor physical role (⇩)	-Low level of general health (⇩) -Low level of education (⇩)
Jafari et al (21)	2012	SF-36	-Sports activities (⇧) -Aging (⇩)	-Psychological problems (⇩) -Low level of general health (⇩) -High level of education (⇧) -Employment (⇧)-Number of children (⇩)
Mousavi et al (22)	2011	SF-36	-Chronic pain (⇩) -Disturbance of physical role (⇩) -Lack of daily activities (⇩)	-Poor emotional role (⇩) -Low level of general health (⇩) -Work problems (⇩) -Disorders of interpersonal relationship (⇩)
Attaran et al (23)	2006	SGRQ	-One stage increase in spirometric parameters (⇩) -Decrease in FEV1 (⇩) -Severity of chemical injury (⇩)	-Psychological problems (⇩)
Arefnasab et al (24)	2013	SGRQ	-Severity of chemical injury (⇩)	-Deterioration of mental health (⇩) -Depression (⇩) - Anxiety (⇩) -Disturbance of social functioning (⇩)
Mehdizadeh et al (25)	2011	SGRQ	-Co-existence of pulmonary, ocular, dermal and mental disorders (⇩) -Severity of chemical injury (⇩) -Decrease in FEV1 (⇩)-Bronchiolitis (⇩)	-Quality of sleep (⇧) -Psychological problems (⇩) - High level of education (⇧) -Employment (⇧)
Tavallaie et al (26)	2006	SGRQ	-Co-existence of pulmonary, ocular, dermal and mental disorders (⇩), -Bronchiolitis (⇩)	-Psychological problems (⇩) -History of taking psychiatric medicine (⇩) -History of hospitalization for psychiatric complications (⇩) -Educational level (⇧) - Employment (⇧)
Tavalie et al (27)	2008	W-QLI	-Poor general health (⇩)	-Psychological problems (⇩) -Inadequate salary (⇩)
Panahi et al (28)	2008	DLQI	-Severity of pruritus (⇩) -Burning sensation (⇩) -Blistering (⇩) -Lichenification(⇩) -Excoriation (⇩) -Relieving skin lesions (⇧)	-Sexual disorders (⇩) -Low level of symptoms and emotions (⇩) -Disorders of interpersonal relationship (⇩) -Lack of daily activities (⇩)
Biat Saeed et al (29)	2104	Ebadi’s Specific Tool	-Co-existence of pulmonary, ocular, dermal and mental disorders (⇩) -Lengthy continuation of chemical injury (⇩) -Poor physical health (⇩) - Aging (⇩)	-Psychological problems (⇩) -High level of education (⇧)-Job appropriateness (⇧)
Berahmani et al (30)	2004	Berahmani’s specific Tool	-Severity of chemical injury(⇩)	-Employment (⇧) -Educational level (⇧)
		Positive factor (⇧)		Negative factor (⇩)

Moreover, the following instruments were used for the assessment of QoL among the included studies: the Short Form Health Survey (SF-36) in 4 articles (19–22), the St. George’s Respiratory Questionnaire (SGRQ) in 4 articles (23–26), the Wisconsin Quality of Life Index (WQLI) in 1 article (27), Dermatology Life Quality Index (DLQI) in 1 article (28), Ebadi’s specific tool in 1 article (29), Abbasi’s specific tool in 1 article (15) and Berahmani’s specific tool in 1 article (30).

All included articles reported low QoL scores for the mustard gas victims, and several subjects exhibited an extremely poor level of QoL (20–22, 25). Some articles also compared the QoL of mustard gas victims with that of the general population, which revealed a low QoL level among mustard gas victims (20–22, 30). Further, the studies revealed that mustard gas victims experienced further problems in some QoL subscales, i.e., mental health (15, 21, 29), symptoms and activities (23, 25, 26), general health, and physical functioning.

### Physical factors and QoL of mustard gas victims

Among physical factors influencing the QoL of mustard gas victims, concurrent existence of pulmonary, ocular, dermal, and psychiatric complications had the most damaging impact on the QoL of these patients (15, 19, 25, 26, 29). The severity of chemical injury was also a factor that could considerably exacerbate the QoL of chemically injured veterans (23–25, 30), though several studies (19, 26, 29) refuted such a relationship. Besides, some studies suggested that a low level of physical health could account for decline in life quality (19, 27, 29). Accordingly, a number of studies reported that participation in sports activities could be an influential factor in improving the QoL of exposed veterans (20, 21). The persistence of complications due to chemical exposure was the other parameter described in two studies, which impaired the QoL of mustard gas victims (20, 29). Of course, it was a matter of controversy because a few studies discerned no connection between lengthy continuation of chemical complications and the QoL of chemically injured veterans (23, 26). Notably, studies regarding bronchiolitis and spirometer parameters recognized FEV1 as a factor influencing the QoL of mustard gas victims (23, 25, 26). Further, Panahi et al., reported that dermal complications like pruritus, burning sensation, blistering, excoriation, and lichenification significantly reduced the QoL of exposed veterans. Therefore, they recommended treating skin lesions to help these veterans enjoy higher levels of QoL (28). Furthermore, several studies identified the role of poor physical function (20, 22) as well as lack of daily activities (22, 28) as influential factors in the decline of QoL, while another study defined chronic pain (22) as a parameter impairing the QoL of chemically injured veterans. The impact of aging on the QoL of mustard gas victims was also deemed important; however, not all studies confirmed the same. Besides, articles by Jafari et al. and Biat Saeed et al. (21, 29) showed a link between age and QoL, which was disproved by Attaran et al. and Tavallaie et al. (23, 26).

### Psychosocial factors and QoL of mustard gas victims

The results of the current systematic review indicated that mental complications were some of the topmost factors decreasing the QoL of mustard gas victims (21, 23, 25–27, 29). In addition, a low level of general health proved to be an influential factor (19–22). In fact, deterioration in mental health, depression (24), and anxiety (15, 24) lowered the QoL of chemically injured veterans. Other factors that reduced the QoL of victims included poor emotional role (19, 22), sexual disorders (28), history of taking psychiatric medication, history of hospitalization for psychiatric complications (26), low level of mood and emotions (28), and destruction of self-confidence (15). On the other hand, higher levels of spirituality and religious beliefs (19), along with good quality of sleep (25), helped these patients enjoy better QoL. Furthermore, the results of the current systematic review revealed that mustard gas victims with higher educational levels had better QoL (20, 21, 25, 26, 29–31). Additionally, factors of employment and job appropriateness to the special condition of chemically injured veterans played positive roles in enhancing the QoL of these patients (21, 25, 26, 29, 30). Some studies recognized work problems (22) and inadequate salary (27) as factors that reduce QoL. Further, disorders of interpersonal relationships and social functioning (22, 24, 28) were identified as other parameters that lowered QoL. Finally, Jafari et al. (21) reported that increase in the number of children could influence QoL, while a study by Biat Saeed et al. (29) refuted such a connection.

## DISCUSSION

Several chemically injured veterans suffer from a poor level of QoL though it has been 30 years since the first exposure to mustard gas (32). Therefore, the current systematic review aimed to determine the physical and psychosocial factors that affect the QoL of mustard gas victims. To our knowledge, this was the first systematic review that investigated the QoL of mustard gas attack survivors.

Thus far, there have been several attempts to gather comprehensive information on the life experiences of exposed veterans, but such studies have failed to develop a decisive and well-rounded protocol for planning clinical care and psychological services for chemically injured veterans. The current systematic aimed to lay foundations for research and psychosocial intervention to improve the QoL of exposed veterans.

Given that mustard gas victims experience particularly distinct aspects of life and they have to deal special problems, it is important to consider diagnoses and care plans fitting their condition (12).

Mustard gas victims sustain several medical conditions (pulmonary, dermal, ocular, neurological, and gastrointestinal disorders) in addition to psychiatric distress. Therefore, their QoL can be assessed from different aspects. Such a multi-dimensional nature also entails using specific instruments that are only designed for mustard gas victims. Considering these factors, major improvements in the QoL of exposed veterans can be achieved by taking small steps to change their lifestyle (33).

The results of the current systematic review also indicated that despite the availability of literature on chemically injured veterans, some details and facts are missing in over half of the studies. Such details and facts mostly related to important parameters such as duration of suffering from chemical injury, intensity of chemical injury, time of mustard gas exposure, and underlying disorders and comorbidities. In fact, several studies assessing the QoL of mustard gas victims reported that these parameters play a significant role (15). Such data profoundly influences the rate of veterans’ response to treatment. Further, it could be divided into two categories, namely, internal factors, including health status and underlying genetic disorders, and external factors like smoking, exposure to other agents, and frequency of exposure (34).

The current systematic review indicated that, among the physical factors affecting the QoL of mustard gas victims, the most frequently identified influence was the co-existence of several diseases such as pulmonary, dermal, ocular, and psychiatric complications (15, 19, 25, 26, 29). This factor was followed by the severity of chemical injury, poor physical health, and pulmonary problems, respectively. In fact, a low level of physical health, as reported in several studies (19, 27, 29), along with suffering from long-term complications of mustard gas (35, 36), can be deemed as the most influential factor lowering the QoL of exposed veterans. Several studies heavily stressed that the co-occurrence of several diseases; other factors such as aging (21, 29), long persistence of mustard gas complications (20, 29); and severity of chemical injury, together with the number of damaged organs (23–25, 30) can largely impact the QoL of chemically injured veterans. As these patients face several physical problems, they usually suffer from poor physical functioning as well (20, 22), which often leads to obesity and diabetes, and consequently, results in the reduction of QoL (21, 37). Furthermore, findings of the included studies showed that the long-term complications of mustard gas increased the risk of developing cancer (15, 19). Recent studies also highlighted the risk of cancer due to mustard gas even years after exposure (38–40). Results of a study by Ghanei et al. reflected that pseudo-cancerous symptoms that may occur due to brief exposure to mustard gas still heightened the risk of cancer in exposed veterans (39). A cohort study by Zafarghandi et al. also indicated that the prevalence of cancer among those exposed to mustard gas was remarkably high (40). Further, cancer patients who are treated using mustard compounds ultimately develop secondary malignant growths like Leukemia and Lymphoma (12, 41). Studies on cancer also suggest that the QoL of cancer patients drastically decreases during the course of the illness (42). As a result, exposed veterans’ QoL is impaired, first by the late complications of the chemical agent, and then by the fear of developing cancer (15, 19). However, plans to psychosocially support these patients can favorably influence their QoL, as well as that of their families (43).

Among the psychosocial parameters affecting the QoL of mustard gas victims, the following influencing factors were identified: psychological problems (21, 23, 25–27, 29), educational level (19–21, 25, 26, 29, 30), and employment and job appropriateness to the special condition of chemically injured veterans (21, 22, 25–27, 29, 30). These factors were followed by low level of general health (19, 21, 22, 24, 30), disorders of interpersonal relationships and social functioning (22, 24, 28), and depression and anxiety.

It is probable that the sample group suffered from impaired QoL due to physical and mental disorders (35, 36, 44), as well as due to problems such as having a history of hospitalization for psychiatric complications or taking psychiatric medication (26), poor emotional functioning (19, 22), low level of mood and emotions (28), destruction of self-confidence (15), sleep disturbances (25) and sexual disorders. Notably, chronic diseases and psychological problems often lead to adverse effects on sexual function (45). In this regard, Ranjebar Shayan et al. (45) revealed that 65.4% of mustard gas victims suffered from some type of sexual dysfunction. Panahi et al. (28, 46) also reported that sexual relations, which constituted a facet of QoL, was more strongly influenced by mustard gas exposure as compared to other problems. Abbasi (15)also demonstrated that over half of the chemically injured veterans were strongly influenced by the dermal complications caused by mustard gas (22, 24, 28). Therefore, conditions such as pruritus and burning sensation may cause victims’ relatives and acquaintances to feel uncomfortable and to deem the conditions as disgraceful when communicating with mustard gas victims. Even the veterans are likely to presume that others experience such feelings. In fact, a sudden irresistible sensation of itch in different parts of body which are mostly difficult to scratch, especially the groin, turn pruritus a serious problem for exposed veterans (28, 46). Additionally, mustard gas victims suffer from pulmonary diseases that are accompanied by expulsion of sputum, persistent coughing, wheezing, and shortness of breath (47). When these symptoms appear, in most situations, they provoke negative feedbacks and finally lead to social isolation, depression, anxiety, sleep disorders, and disturbances of interpersonal relationships among exposed veterans (48). Thus, when mustard gas complications occur over a long period, patients often exhibit aggression, anger, and reluctance to engage in social interactions, which together impair their QoL (49). However, recent studies strongly emphasize on the importance of social support for treating the mental and even physical complications of chemically injured veterans (43, 50).

In most studies, a high level of education was reported to have a positive influence on the QoL of injured veterans. This may be explained by the fact that the increase in knowledge about methods of symptoms control, together with rise in social interactions, may help improve QoL (20, 21, 29). Moreover, employment and economic problems stemming from medical expenses played key roles in the QoL of mustard gas victims (15, 22), in addition to several other problems, particularly the lack of daily activities. It is also notable that health care costs constituted a major item of their expenditure (51, 52). Generally, it has been reported that mustard gas victims can hardly handle personal affairs and almost all had problems related to managing living costs (22, 51) because they not only sustained physical disorders, i.e., pulmonary (21, 53), dermal (28, 46), and ocular (22) complications, but they also experienced fatigue (54, 55), sleep disturbances (56) and adjustment disorders (44). Further, they suffered from mental diseases, e.g., depression, anxiety, and PTSD (10, 35, 57–59). Abbasi (15) reported that exposed veterans often encountered unsuitable income level, employment status, and dwelling place. This explains why veterans with an appropriate job enjoyed superior QoL, as employment substantially improves the QoL of mustard victims (21, 25, 26, 29, 30). Inability to afford living costs can lead to physical and mental complications, and in turn, impair QoL (27, 60). However, in sum, it is clear that most studies have failed to suggest a holistic approach to intervention. In fact, given their special physical and psychosocial conditions, exposed veterans need comprehensive plans and measures to enhance their QoL.

One of the constraints that the current review encountered was that though the studies scrutinized herein contained a huge mass of data on veterans’ QoL, some pieces of information, such as mean time since last exposure, frequency of exposure, and severity of chemical injury were neglected. Hence, this study recommends that prospective studies should make use of specific instruments to evaluate the QoL of mustard gas victims and should address the above-mentioned variables.

## CONCLUSION

The analysis conducted within this review backs up the theories on the impact of physical and psychosocial factors on the QoL of exposed veterans. The findings suggest that the coexistence of several medical conditions caused by the chemical injury, severity of chemical injury, psychological and neurological complications, educational level, and employment status and job-appropriateness had the greatest impact on the QoL of mustard gas victims. Nevertheless, long-term studies are required to determine the correlation between veterans’ QoL and these physical and psychosocial parameters. In future, research should focus on improving the QoL of these patients by assembling a multi-disciplinary medical team.
